# Supplementary material for: Personalised Dosing Using the CURATE.AI Algorithm: Protocol for a Feasibility Study in Patients with Hypertension and Type II Diabetes Mellitus
Source: Int J Environ Res Public Health. 2022 Jul 23;19(15):8979. doi: 10.3390/ijerph19158979 (PMC9332044; doi:10.3390/ijerph19158979)
Supplement: Supplementary file 1 [file ijerph-19-08979-s001.zip › ijerph-1795625-supplementary.pdf]

Outpatient Experience Survey

Personalised dosing in patients with hypertension or type II diabetes

Participant characteristics

1. Date of visit
2. Are you the: Patient or caregiver,
3. Residency status: Singaporean, permanent resident,
4. Age (years):
5. Employment status: Employed, unemployed, retired, homemaker
6. Long-term conditions:

Clinic care experience

7. Did the doctor explain the reasons for any treatment or action in a way that you could understand?

[ ] Yes, completely
[ ] Yes, to some extent
[ ] No

8. Did the doctor listen to what you had to say?

[ ] Yes, definitely
[ ] Yes, to some extent
[ ] No

9. Did you have confidence and trust in the doctor examining and treating you?

[ ] Yes, completely
[ ] Yes, to some extent
[ ] No

10. Did your appointment help you to feel that you could better manage your condition or illness?

[ ] Yes, definitely
[ ] Yes, to some extent
[ ] No

11. Overall, did you feel you were treated with respect while you were at the Clinic?
[ ] Yes always
[ ] Yes sometimes
[ ] No

Remote-care experience

12. Did you feel confident using equipment to self-monitor your condition? (e.g. blood pressure machine)
[ ] Yes, definitely
[ ] Yes, to some extent
[ ] No

13. Was self-monitoring easy to fit into your routine life?
[ ] Yes, definitely
[ ] Yes, to some extent
[ ] No

14. Were issues with self-monitoring equipment easily resolved by staff?
[ ] Yes, definitely
[ ] Yes, to some extent
[ ] No

CURATE.AI concept

15. Do you have confidence in the CURATE.AI system to correctly dose your medication?
[ ] Yes, definitely
[ ] Yes, to some extent
[ ] No

16. Do you think the CURATE.AI system benefits you as a patient and in what ways?

17. Do you think the CURATE.AI system benefits healthcare professionals and in what ways?

18. What, if any, were the challenges of using the CURATE.AI system?

19. What, if anything, could be improved?

20. Do you have any further questions?
